# Critical analyses of Latina mortality: disentangling the heterogeneity of ethnic origin, place, nativity, race, and socioeconomic status

**DOI:** 10.1186/s12889-024-17721-9

**Published:** 2024-01-16

**Authors:** Vanessa L. Errisuriz, Ruth Enid Zambrana, Deborah Parra-Medina

**Affiliations:** 1 Santa Clara University, 500 El Camino Real, Santa Clara, CA 95053 USA; 2 Harriet Tubman Department of Women, Gender and Sexuality Studies, University of Maryland, Susquehanna Hall 4200 Lehigh Rd. Room 4117, College Park, MD 20742 USA; 3 Latino Research Institute, University of Texas at Austin, 210 W. 24th Street, GWB 1.102, Austin, TX 78712 USA

**Keywords:** Latina health, Mortality, Leading causes of death, Chronic disease

## Abstract

Despite the significant body of research on social determinants of health (SDH) and mortality, limited knowledge is available on the epidemiology of aggregated Latino health overall, and by women and subgroups. In population health studies, U.S. Latinos often are considered a monolithic population and presented as an aggregate, obscuring the diversity and variations within and across Latino subgroups, contributing to missed opportunities to identify SDH of health outcomes, and limiting the understanding of health differences. Given diverse environmental, racial, class, and geographic factors, a specific focus on women facilitates a more in-depth view of health disparities. This paper provides a scoping review of current gaps in research that assesses the relationships between SDH and mortality rates for the five leading causes of chronic-disease related deaths among Latinas by ethnic origin, place, race, and SES. We analyzed 2020 national mortality statistics from the CDC WONDER Online database jointly with reviews of empirical articles on Latina health, employing the EBSCOhost MEDLINE databases. These findings challenge the phenomenon of the Hispanic paradox that identified Latinos as a relatively healthy population compared to non-Hispanic White populations despite their lower economic status. The findings confirm that prior research on Latino women had methodological limitations due to the exclusion of SDH and an overemphasis on culturalist perspectives, while overlooking the critical role of socioeconomic impacts on health. Findings indicate major knowledge gaps in Latina mortality by SDH and subgroups that may undermine surveillance efforts and treatment efficacy. We offer forward-looking recommendations to assure the inclusion of key SDH associated with Latina mortality by subgroup as essential to inform future studies, intervention programs, and health policy.

**Supplementary Information:**

The online version contains supplementary material available at 10.1186/s12889-024-17721-9.

## Introduction

In the United States (US), a substantial body of population health research exists on Hispanics/Latina/os (hereafter Latinos) as Latinos are disproportionately burdened by disease and death. In particular, Latinos have a higher prevalence of obesity (45.6%) than non-Latino Whites (41.4%) [1], which is concerning given that obesity traits (e.g., waist-to-hip ratio, body mass index) are causally linked to the majority of the leading causes of death from non-communicable (i.e., chronic) diseases [2]. About half of the U.S. Latino population self-identify as women (i.e., Latinas), making up 18.9% of all women in the US [3]. Current demographic trends suggest that Latinas will represent 28.6% of the total female population by 2060 [4]. To attain a comprehensive understanding of Latino health disparities, it is important for researchers to consider sex differences, given that health and illnesses present and are experienced differently by men and women. For instance, women are more likely than men to report poorer self-rated health, disability, and life-threatening medical conditions [5]. Research that examines factors that lead to mortality disparities in chronic diseases among Latinas can provide further clarity for solutions and inform health policy.

ITo better elucidate Latina health disparities, researchers should consider the heterogeneity of the population. Although Hispanics tend to be treated as a monolithic or homogeneous population, the designation refers to native- or foreign-born individuals who are or have ancestors from over 20 countries where Spanish is mainly spoken, including those people from the Caribbean, Central America, and South America [6–8]. In 1977, the term *Hispanic* was federally codified by the Office of Management and Budget and subsequent collection of Hispanic/Latino ethnicity has become a standard part of demographic data-gathering [9]. However, this all-encompassing classification lumps together Latinos who are both U.S.- and foreign-born, and who are descendants of African, European, and Indigenous populations, and other diasporic populations (i.e., such as Asians).

Data from the U.S. Census Bureau 2021 American Community Survey [10] show stark differences among key social determinants of health indicators among the U.S. Latina subgroups, ranging from education to health insurance access (see Table 1). For example, the percentage of college graduates among South American (39.4%) and Cuban (30.7%) women is 2 to 3 times higher than among Mexican American (14.8%) and Central American (15.6%) women. Latinas of South American origin are also more likely to be employed and have health insurance and are less likely to live below the poverty line compared to Central American- and Mexican-origin women. These demographic profiles suggest that health outcomes likely differ substantially by ethnic origin among Latinas, but the dearth of research on health outcomes among Latina subgroups has resulted in inadequate data for investigating within- and between-group disparities [11, 12].

**Table 1:** Sociodemographic characteristics of U.S.Latinas by subgroup in 2021 (%)

	≥18 years	High school or higher	Bachelor’s degree or higher	≥16 years employed	Below poverty^a^	No health insurance^a^	U.S.-born	Foreign-born, naturalized citizen	Foreign-born, not U.S. citizen
Latina overall	49.7	73.9	21.6	55.2	17.5	17.7	68.1	13.0	18.9
Mexican	49.0	69.8	16.9	54.7	17.5	19.5	71.3	9.9	18.7
Puerto Rican	50.9	84.1	25.9	53.3	20.3	8.0	97.7	1.2	1.1
Cuban	49.4	82.8	32.0	55.0	14.1	12.2	46.6	35.4	18.0
Dominican	54.4	74.6	24.4	56.2	20.0	9.7	49.6	28.1	22.3
Central American	48.8	63.0	17.7	56.9	19.7	28.2	44.7	18.4	36.9
South American	52.4	88.2	40.0	60.2	12.1	14.1	40.8	32.5	26.7
Other Latina	50.8	82.5	27.5	54.2	18.3	9.6	78.3	12.8	8.9

SOURCE: United States Census Bureau. American Community Survey ACS 1-Year Estimates Selected Population Profiles 2021. Census.gov. Accessed January 5, 2023

^a^ % estimate includes male and female Latinos

### Social determinants of Latina health

Social determinants of health (SDH) is a construct that encompasses the myriad external conditions in which people are born, live, learn, work, reproduce, and age, and the expansive set of institutional forces and systems that affect those conditions [13]. The SDH, alone or in combination, shape material and psychosocial conditions that may contribute to the manifestation and escalation of numerous life-threatening health problems, including cardiovascular disease, diabetes, and cancer. Aggregating women from diverse geographic, cultural, social, racial, and historical backgrounds into one demographic classification (i.e., Latinas) masks important differences and erases opportunities to distinguish specific factors associated with morbidity and mortality by subgroup. The Healthy People Initiative objectives highlight national health priorities, provide measurable goals, and monitor progress towards achieving goals and improving the health of the nation [14]. Over time, the Healthy People Initiative has revised and strengthened its language so that eliminating health disparities, achieving health equity, and creating physical, social, and economic environments are clear priorities [14]. Although there is growing awareness of the need to decrease health inequities, eliminate health disparities, and create approaches that combine systemic efforts from education, housing, health care, and other sectors, possible paths have not been mapped out and have not resulted in steps forward to advance health equity [15].

Over the life course, accumulated exposure to multiple socioeconomic social determinants shape Latinas’ state of health and illness [16–18]. A recent systematic review of public health surveillance surveys in the US found that only 30.5% of articles focusing on Latino health further disaggregated Latino child and adult health estimates by key SDH such as race, nativity, SES, and place [19]. Engaging an intersectional lens to critically examine factors that affect Latina health provides a useful sensitizing framework for understanding how multiple forms of structural disadvantage jointly influence health outcomes.

We employed this critical lens to examine how demographic characteristics (e.g., race, ethnicity, education level) often serve as markers of inequity and disparity. We assess the current state of the science on Latina health by looking closely at the relationships between SDH (i.e., ethnic origin, place, nativity, race, and SES) and mortality rates for five prevalent chronic conditions (e.g., cancer, heart disease, cerebrovascular disease, type II diabetes and Alzheimer’s). We conclude with critical consensus points aligned with the literature, identify SDH-related gaps, and provide forward-looking recommendations to advance improvements in Latina health.

## Methods

We utilized the conceptual framework developed by the Commission on Social Determinants of Health (CSDH), which was established by the World Health Organization, to synthesize SDH-related evidence into one framework and highlight key differences between levels of causation (i.e., mechanisms through which social hierarchies and resulting daily life conditions occur) [13]. The CSDH framework posits that social contexts such as the structure or social relations of society lead to social stratification (e.g., by race, gender, class), which in turn leads to differential exposures, vulnerability to, and consequences of ill health. For this review, we focus on important social stratifiers identified by the CDSH: gender, race/ethnicity, SES, and nativity as well as place, an important intermediary determinant of health, that links social stratifiers to differential health outcomes.

Similar to previous work reviewing Latino health in the US [20], we used Arskey and O’Malley’s scoping methodology [21] to examine Latina adult health over the past two decades. This methodology is useful for reviewing the extant literature when the research question is not well defined, addressing broader topics where a variety of study designs may be included, identifying gaps in the research base, and summarizing and disseminating research findings to stakeholders [21]. A scoping review is similar to a systematic review, however it does not usually involve quality assessment and findings are reported in a narrative format [22].

*Identifying the research question.* The research question we pursue in this review is: What are the relationships between SDH and mortality rates for the most prevalent chronic conditions among Latinas in the US? We used 2020 data from the Centers for Disease Control and Prevention (CDC) WONDER (Wide-Ranging Online Data for Epidemiological Research) database [23] to retrieve the most recently published data on the five leading causes of chronic disease related death among Latinas (i.e., cancer, cardiovascular disease, cerebrovascular disease, Alzheimer’s disease, and type II diabetes). We then searched for studies examining mortality rates for the five leading chronic diseases among US Latino populations that either specifically focused on Latinas or disaggregated data by sex.

*Identify relevant studies*. We identified relevant studies, specifically searching the literature for comprehensive reviews of Latino/a health research that address our question. The initial search was performed by one of the authors with the assistance of a research assistant and librarian with extensive knowledge related to using literature databases. In EBSCOhost Medline, we used the Boolean search terms “Hispanics OR Latinos,” “mortality OR mortality rate OR death OR death rate, “women OR females OR woman OR female,” “race OR ethnicity OR minority,” “social determinants OR health determinants OR determinants”, and then the outcome of interest (e.g., cancer, cardiovascular disease, etc.). A search was conducted separately for each leading chronic disease, and each search was limited to publication date between January 2000 and January 2023, English-language only, and peer-reviewed articles.

The initial searches yielded too few articles to review for citations for Alzheimer’s Disease (*n* = 2), Type 2 diabetes (*n* = 5) and cerebrovascular diseases (*n* = 18) and too many articles tangential to our research question. A more targeted approach (Table 2) resulted in 361 articles that were more specific to our research question. We also utilized lateral search techniques such as performing key word searches in Google Scholar, checking reference lists of systematic, narrative, scoping, or literature reviews, and examining publications produced by PEW Research Center, the Study of Women’s Health across the Nation, the Hispanic Community Health Study/Study of Latinos, and the Women’s Health Initiative. Previous research has demonstrated that lateral search strategies may be particularly important for identifying non-randomized studies [22].

**Table 2:** Search strategy used in EBSCOhost medline

Search Component	Search Terms
Cardiovascular Disease	latinos or hispanics (abstract) AND cardiovascular disease or cvd or heart or cardiac or coronary heart disease (abstract) AND mortality or mortality rate or death or death rate (title) AND women or female or woman or females (all text) AND race or ethnicity or minority
Cancer	latinos or hispanics (abstract) AND cancer AND mortality or mortality rate or death or death rate (title) AND women or female or woman or females (all text) AND race or ethnicity or minority AND united states (no field selected)
Cerebrovascular Disease	latinos or hispanics (abstract) AND cerebrovascular disease stroke (all text) AND mortality or mortality rate or death or death rate (title) AND women or female or woman or females (all text) AND race or ethnicity or minority (all text) NOT cardiovascular disease or cvd or heart or cardiac or coronary heart disease
Alzheimer’s Disease	latinos or hispanics (abstract) AND Alzheimer’s disease (all text) AND mortality or mortality rate or death or death rate (title) AND women or female or woman or females (all text) AND race or ethnicity or minority
Diabetes	latinos or hispanics (abstract) AND diabetes type 2 or diabetes mellitus type 2 or diabetes 2 (all text) AND mortality or mortality rate or death or death rate (title) AND women or female or woman or females (all text) AND race or ethnicity or minority

The initial EBSCOhost MEDLINE database search yielded 361 articles, and we identified an additional 12 articles from the lateral search for a total of 373 articles to screen. We removed 6 duplicate articles, resulting in 367 articles to screen. Of these, 221 articles were excluded due to either a lack of focus on Latinas or not including the primary outcomes of interest (e.g., chronic disease-specific mortality rates). We assessed a total of 146 full-text articles for eligibility. Table 3 shows the breakdown of the literature search by chronic disease.

**Table 3:** EBSCOhost MEDLINE and lateral search articles on mortality rates among hispanic women or Latina subjects, by chronic disease (2000–2023)

Chronic Disease	Results Found	After Title/Abstract Screening	After Eligibility Review
Cardiovascular Disease	154	48	12
Cancer	123	71	32^a^
Cerebrovascular Disease	34	12	4
Alzheimer’s Disease	39	10	3
Diabetes	17	5	1
Total:	367	146	52

Search conducted February 9, 2023

^a^ Six articles included another leading chronic disease in addition to cancer

### Study selection and charting the data

**Fig. 1:**
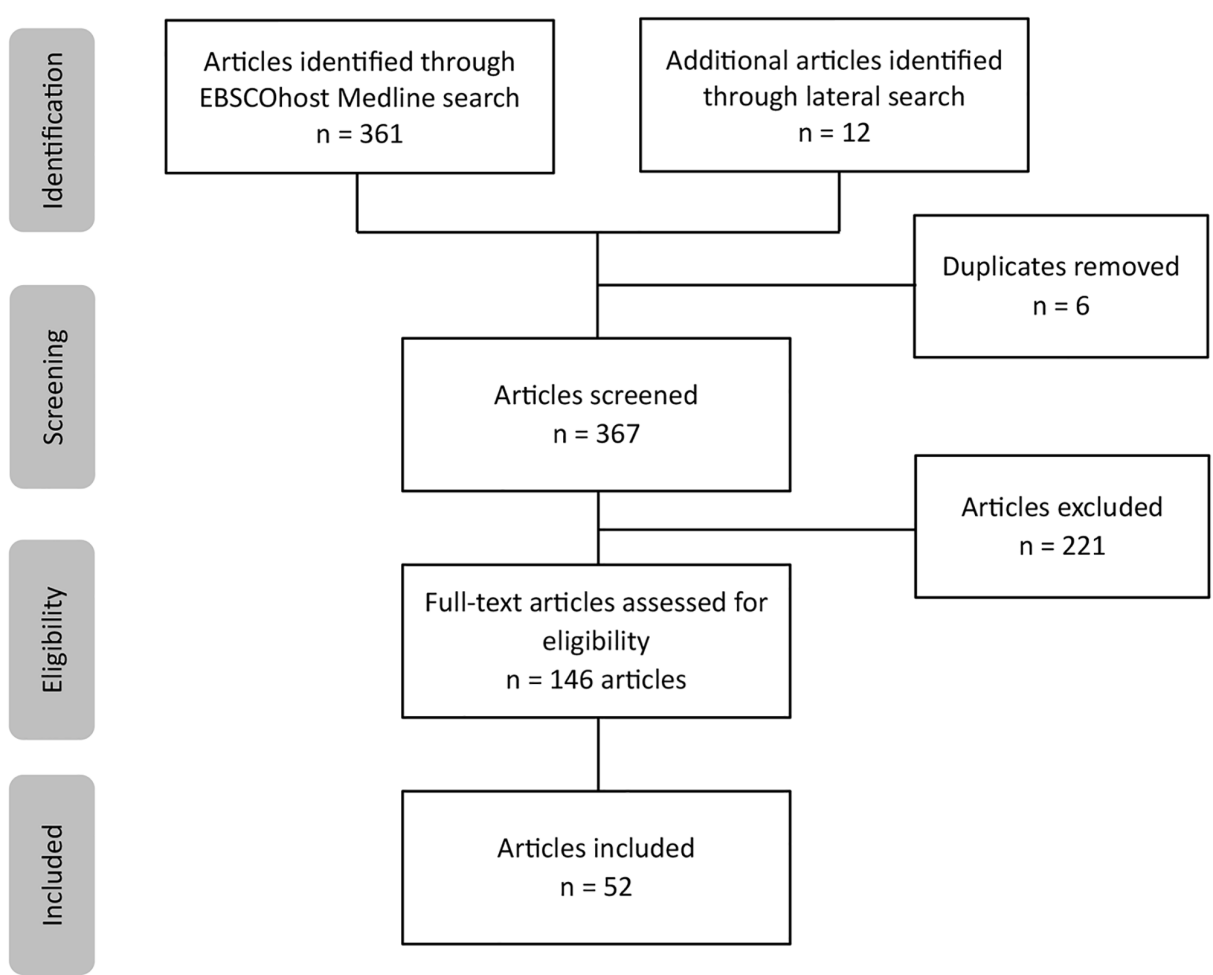
Literature review

The initial reference list containing 146 articles was exported into Excel and reviewed by one author and a research assistant. This resulted in 95 exclusions due to lack of focus on mortality rates for at least one of the leading five chronic diseases among U.S. Latinas and/or not reporting gender-disaggregated outcomes for Latinas. The final sample consisted of 52 articles (See Fig. 1). Data extracted from the article included title, author, and year of publication. We also identified whether data were further disaggregated by ethnic origin, nativity, race, SES, and place (see supplemental Table 1).

### Reporting the results

Review of the articles to be included showed that no studies disaggregated Latina mortality data by race. As indicators of socioeconomic status are highly intertwined with race [24, 25], studies were grouped by SDH into the following categories: (1) ethnic origin, (2) place, (3) nativity, and (4) race and socioeconomic status. Additionally, very few studies disaggregated mortality data by place, and those that did focused on cancer. We used the CDC WONDER database to supplement our critical analysis and examine age-adjusted mortality rates in 2020 for the five leading causes of chronic disease-related death among Latinas who reside in the five U.S. states with the highest Latino populations (i.e., California, Texas, Florida, New York, and Arizona). All deaths related to the five leading causes of chronic disease-related death among Latina and non-Latina White females (all ages) were included. Data for deaths related to these diseases were obtained by querying “diseases of circulatory system” using International Classification of Diseases-10th Revision (ICD-10) codes I00–I09, I11, I13, and I20-I51; “cancer” using ICD-10 codes C00-C97; “cerebrovascular disease” using ICD-10 codes I60-I69; Alzheimer’s disease using ICD-10 code G30; and “diabetes mellitus” using ICD-10 codes E10-E14 as contributing or underlying causes of death. Each disease was queried separately for Hispanic or Latino females of all races and White females who were not Hispanic or Latino. All ages were included in the queries, and we grouped results by the five aforementioned states. Finally, we examined age-adjusted mortality rates (AAMR) per 100,000 population, calculated by standardizing the deaths to the year 2000 U.S. population.

## Results

### Social determinants of health and leading mortality indicators among U.S. Latinas

In 2020, the top five chronic disease related leading causes of death among U.S. Latina adults, after adjusting for age, were (in order): cardiovascular disease, cancer, cerebrovascular disease, Alzheimer’s, and diabetes. Among Latinas, cardiovascular disease and cancer were the leading causes of death, similar to non-Latino White (NLW) women. Cerebrovascular diseases and diabetes ranked higher as leading causes of death among Latinas than NLW women [23].

Research findings have consistently shown that, compared to NLW counterparts, Latinos have lower overall mortality rates for chronic diseases such as cardiovascular disease [26, 27] and almost all cancers [28, 29], although they are at greater risk for both. CDC data demonstrate similar patterns of mortality rates for Latinas relative to NLW women across all five leading causes of chronic disease-related death (See Table 4). However, increasing evidence suggests that mortality rates for both cardiovascular disease and cancer differ by Latino subgroup including ethnic origin, place, nativity, and race [26, 30–32].

**Table 4:** Age-adjusted mortality rates among Latinas and Non-Latina white women overall and by statea

	Cardiovascular Disease	Cancer	Cerebrovascular Disease	Alzheimer’s Disease	Diabetes
**Overall**					
Latinas	100.2 (98.6–101.7	92.8 (91.3–94.3)	34.1 (33.2–35.0)	35.2 (34.2–36.1)	26.7 (25.9–27.5)
Non-Latina White	124.6 (123.6–125.6)	124.6 (123.6–125.7)	35.8 (35.3–36.3)	36.7 (36.2–37.2)	13.7 (13.4–14.1)
**Arizona**					
Latinas	90.9 (83.9–97.8)	97.4 (90.6–104.3)	32.8 (28.6–37.0)	38.1 (33.4–42.8)	31.6 (27.6–35.5)
Non-Latina White	114.4 (111.1–117.7)	115.6 (111.9–119.2)	31.1 (29.4–32.9)	38.3 (36.5–40.2)	14.3 (13.0–15.6)
**California**					
Latinas	94.0 (91.4–96.5)	97.4 (94.8–99.9)	33.5 (32.0–35.0)	39.7 (38.0–41.4)	29.9 (28.4–31.3)
Non-Latina White	119.1 (117.3–120.9)	125.8 (123.8–127.8)	38.3 (37.2–39.3)	52.7 (51.6–53.9)	13.5 (12.9–14.2)
**Florida**					
Latinas	90.1 (86.9–93.2)	86.3 (83.1–89.4)	41.9 (39.8–44.1)	28.3 (26.5–30.0)	17.0 (15.6–18.4)
Non-Latina White	109.6 (107.7–111.5)	126.4 (124.2–128.6)	42.0 (40.9–43.1)	22.4 (21.6–23.1)	13.3 (12.6–14.0)
**New York**					
Latinas	122.8 (117.8–127.8)	80.4 (76.3–84.4)	21.0 (18.9–23.0)	14.9 (13.2–16.7)	18.8 (16.8–20.8)
Non-Latina White	139.9 (137.6–142.2)	123.2 (120.9–125.6)	22.5 (21.6–23.4)	17.2 (16.4–17.9)	13.1 (12.3–13.8)
**Texas**					
Latinas	107.0 (103.8–110.3)	95.9 (93.0–98.9)	33.1 (31.2–34.9)	45.1 (42.9–47.3)	32.8 (31.0–34.6)
Non-Latina White	140.7 (138.4–143.1)	127.6 (125.3–130.0)	39.8 (38.6–41.1)	55.1 (53.7–56.6)	14.9 (14.1–15.7)

^a^ Only the five U.S. states with the highest Latino populations were included

### Ethnic origin

Although there is scarce research investigating health outcomes among Latinas, the existing data reveal significant differences in mortality rates within Latina ethnic subgroups [11, 12]. Rodriguez and colleagues [33] used the U.S. National Center for Health Statistics mortality records to examine rates of cardiovascular disease among the three largest Latino subgroups in the US (i.e., Mexican, Puerto Rican, and Cuban). Cuban and Puerto Rican women had the highest proportional mortality rate ratios for ischemic heart disease compared to Mexican and NLW women, across the age spectrum. Although overall Latinas have lower mortality rates for cardiovascular and ischemic heart disease than NLW women, disaggregated data indicate mortality rates among Puerto Rican women to be more similar to NLW women than to other Latino subgroups [33].

Similar to cardiovascular disease, Latinas have lower cancer mortality rates relative to their NLW counterparts. However, disaggregated data demonstrate important disparities within Latino subgroups. For example, women of Mexican, Puerto Rican, and Cuban origin exhibit higher breast cancer mortality rates than Central and South American origin women [34]. Compared to NLW, Puerto Rican women have higher mortality rates for cervical and liver cancers, and Cuban women experience higher mortality burden for endometrial and colorectal cancer [30, 35]. In California, Puerto Rican and Cuban women demonstrated higher incidence of colorectal and lung cancers than Mexican women [36, 37].

There also is evidence suggesting that specific Latino ethnic subgroups exhibit similar rates of stroke compared to NLW. Mexican women exhibit higher rates of cerebrovascular disease deaths compared to Cuban and Puerto Rican women, and these rates are comparable to those of NLW women [33]. In comparing cerebrovascular disease mortality rates, Cuban women experienced the lowest rates compared to Mexican, Puerto Rican, and NLW women. These findings are consistent with earlier studies conducted with Latinos, although the data were not analyzed by gender [38, 39].

A review of Alzheimer’s research among Latino populations estimate a 0.8% incidence among Mexicans living in California, while incidence among Caribbean Latinos in North Manhattan ranges from 2.3 to 5.3% [40]. National data on differences in Alzheimer’s rates by Latino ethnicity does not disaggregate the data by sex, limiting ability to interpret these findings. For diabetes, mortality rates are higher among Latinas relative to NLW women [41], yet there is limited research disaggregating diabetes mortality rates by sex or ethnic subgroup with much of the previous work primarily cross-sectional, centered on one Latino ethnic group, or focused on one region of the US [41]. Diabetes mortality among U.S. Latinos (i.e., including women and men) of Mexican origin is 50% higher than NLWs [41]. Although we were unable to locate disaggregated diabetes mortality rates for Latinas by subgroup, prevalence rate data suggest differences in mortality among Latinas by ethnic group. Mexican and Puerto Rican women exhibit higher prevalence rates than NLW women, whereas Central, South American, and Cuban women demonstrate similar rates to NLW women [42], with rates ranging from 9.8% in South American women to 19.5% in Puerto Rican women [43].

### Place

A growing body of research links neighborhood characteristics and the social and physical surroundings of communities to residents’ health conditions and behaviors. Individuals living in socially and structurally disadvantaged neighborhoods (e.g., food deserts, lack of green space) generally report poorer health and dietary habits and less physical activity relative to residents of more advantaged neighborhoods [44–46]. Latinos are more than twice as likely to reside in high-poverty and disadvantaged neighborhoods relative to NLWs [47, 48], which has strong implications for neighborhood conditions, access to resources, and ultimately, health disparities. Few studies disaggregated data by place, and those that did were related to cancer mortality disparities. For instance, among California Latinas, breast cancer mortality was higher among those that live in low SES versus high SES neighborhoods, but only among U.S.-born Latinas, not foreign-born Latinas [49]. A study using data from the Texas Cancer Registry demonstrated higher breast cancer mortality rates for Latinas compared to NLWs, especially in the Southwest region of Texas– an area that lacks adequate access to mammography screening facilities [50].

Due to the lack of research examining Latina mortality by place, we utilized the CDC WONDER database to examine age-adjusted mortality rates for the five leading causes of chronic disease-related death among Latinas and NLWs who reside in one of the five U.S. states with the highest Latino populations (see Table 4). Cardiovascular mortality rates among Latinas differ by place, with rates highest among Latinas living in New York and Texas, relative to California, Arizona, and Florida. Cancer mortality rates were highest among Latinas living in states along the U.S.-Mexico border (i.e., Arizona, California, and Texas) compared to those residing in Florida and New York. This is consistent with recent research that found cancer mortality rates were higher among Latinos living on the U.S.-Mexico border compared to NLW for people 0–34 years old, however this study did not stratify their data by sex [51]. Place also appears to play an important role in cerebrovascular, Alzheimer’s disease, and type II diabetes mortality. For cerebrovascular disease, Latinas from Florida exhibit higher mortality rates than those from Arizona, New York, California, and Texas. Disaggregation of mortality rates for Alzheimer’s disease and diabetes by place show that rates are higher among Latinas living in Arizona, California, and Texas compared to those in New York or Florida.

### Nativity

Nativity has a significant impact on health outcomes among Latinos. U.S.-born Latinos exhibit more adverse health outcomes and higher mortality rates than their foreign-born counterparts [52]. Although scarce research disaggregates cardiovascular and cancer mortality data by sex and nativity, the following patterns are evident for U.S.-born vs. foreign-born Latinos (men and women): U.S.-born Latinos’ prevalence of hypertension, cancer incidence and survival rates, and mortality rates for liver, kidney and colorectal cancer are higher than foreign-born Latinos [49, 53–55]. Another study found significantly higher gall bladder, stomach, and cervical cancer mortality for U.S.- and foreign-born Latinos compared to NLW [56].

Overall, cerebrovascular mortality rates are lower among Latinos compared to their NLW counterparts. Research examining the impact of misreporting of Latino ethnicity, imprecise measurement of cause of death, and omission of nativity on cerebrovascular mortality rate data found that once these factors were adjusted for, mortality rates from subarachnoid hemorrhage among U.S.-born and foreign-born Latinas were higher compared to than NLW rates [57]. However, both U.S.-born and foreign-born Latina mortality rates due to ischemic stroke and chronic effects of stroke remained lower than NLW after adjustment. Additionally, U.S.-born Latinas exhibited higher rates of stroke mortality than NLW women at younger ages for most subtypes of stroke [57]. With regard to Alzheimer’s disease and related dementia, a dearth of research exists in examining differences in mortality rates among Latinas by nativity. However, both U.S.-born and foreign-born Latinas spend a significantly larger proportion of their remaining years with dementia and therefore less cognitively healthy relative to NLW [58].

### Intersections of race and socioeconomic status

Although race is increasingly recognized as an important contributor to Latina health disparities, we did not find any studies that disaggregated mortality data by race among Latinas. The number of Latinos who identify as Black or Afro Latino (i.e., Caribbean or Latin American of African descent) [59] has more than doubled over the past four decades, yet little is known about the role race plays in Latino health [60, 61]. Among Latinos, there is some evidence that health-related outcomes differ between Blacks and Whites, attributed to skin color (i.e., darker versus lighter) and resulting in differing experiences of racism [61]. Black Latinos had higher prevalence of self-reported hypertension and greater odds of reporting fair or poor self-rated health than White Latinos [62–64]. Furthermore, health disparities between Black and White Latinos closely resembles those of non-Latino Blacks and NLWs [63, 65, 66]. Data suggest the need to examine differences in mortality rates by race among Latinas to increase our understanding of the role race plays in mortality.

Substantial evidence exists on the associations between SES and health. Specifically, individuals of low SES have poorer health and experience higher rates of morbidity and mortality compared to their higher SES counterparts [67, 68]. In 2019, 8.7% of Latinas were working poor, compared with 4.5% of NLW women. Fewer Latinas complete secondary and postsecondary education relative to NLW women, with 77.1% of Latinas earning at least a high school diploma and 20.1% a bachelor’s degree in 2021 compared with 91.2% and 36.6% of NLW women, respectively [69]. Of note, Latinas have the lowest rates of earning a high school diploma and bachelor’s degree compared to all other racial/ethnic groups in the US and are more likely to be unemployed and earn less than NLW and Asian women [70]. These income and educational disparities that differ by subgroup limit Latinas’ access to healthy food and affordable housing in neighborhoods that are safe and/or have infrastructure and resources for physical activity. In turn, their ability to make choices about their and their family’s health, including how to obtain and manage health care is constrained by their economic and community resources [67, 71].

This literature review identified a few studies that disaggregated Latina mortality rates by SES and those that did were focused on overall mortality or cancer-specific mortality [72–77]. Findings from the meagre research that does exist suggests a complex relationship between SES and Latina mortality. One study using data from the National Health Interview Survey found that the overall mortality advantage of Latinos is only concentrated in those of lower SES and that this advantage is minimal or nonexistent at higher levels of SES [78]. Differences in mortality by education were generally smaller for Latino groups than for NLW, the exception being Puerto Rican men and women who showed steep declines in mortality as level of education increased, similar to NLWs. Results of studies examining the relationship between cancer mortality among Latinos and SES are mixed. A study among women in Texas found that Latinas from lower SES background were more likely to die from cervical cancer relative to those from middle and high SES backgrounds [72]. In another study, Latinos in the middle SES group exhibited the largest declines in cancer-related mortality between 1990 and 2000 [79], while a study conducted with California Cancer Registry data show Latinas in the lowest SES group were less likely to die from breast cancer than NLWs [80]. Contrarily, a study examining breast cancer-specific survival among women in the San Francisco Bay Area showed no moderating effect of SES on breast cancer mortality among Latinas [81]. Although studies examining Latina mortality rates by SES for the other four chronic diseases (i.e., cardiovascular disease, cerebrovascular disease, Alzheimer’s disease, and diabetes) were not found, a robust body of evidence shows that the health of low-income predominantly Mexican origin and Puerto Rican women is strongly linked to SDH. These social determinants, particularly education and income, intersect with overlapping complex systems in low resource communities of disadvantage, and negatively impact their health. Over the past decades these trends have not changed significantly, which highlights the compounding urgency to address these health disparities and the systems that contribute to them.

### Scrutinizing findings from Latina health research

The expanding discourse regarding SDH has not been accompanied by significant expansion of epidemiologic research regarding mortality rates for major health conditions by SDH among Latinas. Deficiencies in the extant knowledge base on Latina health restricts the applicability of findings and ignores important subgroup differences in risk profiles and health outcomes that ultimately undermine surveillance efforts and treatment efficacy [82]. Characterizations and examinations of the U.S. Latino populations health research can no longer be described without attention to and recognition of demographic characteristics (e.g., race, ethnic origin, education, nativity) [7] and the disaggregation of data by Latino subgroups. These two analytic strategies allow for needed discovery of population differences by socioeconomic status, place, and other dimensions of inequality [11, 83, 84].

Together, the lack of context regarding SDH and the consistent disregard for Latino heterogeneity have resulted in generalized narratives, such as the “Latino/Hispanic health paradox,” [85] which claims that Latinos exhibit longer life expectancy and better health outcomes compared to NLWs, despite Latinos’ high-risk social and economic profile [82], and the “healthy immigrant paradox” [86] which postulates that Latino immigrants have better health outcomes than their U.S.-born counterparts. Explanations of these purported immigrant health advantages often are explained by the selective migration of healthy people to the US and the return migration of less healthy people to their countries of origin in late life (sometimes labeled “salmon bias”), and personal and family health behaviors [86]. The healthy immigrant effect is not unique to Latinos but observed across highly selective immigrant patterns of movement [87, 88]. Narratives such as the Latino health and healthy immigrant paradoxes have detracted from examining factors contributing to mortality rates of leading chronic diseases among Latinas.

Although observed differences in health outcomes among Latinos often are attributed to cultural rather than to structural factors, sporadic use of acculturation measures and overuse of cultural attributions and culturally (in)competent paradigms exclusive of a regional or class context in Latino health research fail to explain health outcomes [89–91]. Other speculations include that residence among ethnic enclaves may contribute to the lower-than-expected mortality rates due to the presence of strong families and social networks providing a protective effect as Latinos reside in a place where healthier cultural habits are maintained and greater social cohesion and support are obtained [92]. The evidence of the protective effect of enclaves on Latino health is mixed, with more recent work demonstrating that Latinos living in U.S. counties with greater ethnic density experience increased cardiovascular disease and breast cancer mortality than those living in lower ethnic density counties [93, 94]. Reported poverty rates range from 12.1% for women of South American background to 20.3% for Puerto Rican women (Table 1). We argue that the dominance of this culturalist perspective overemphasizes the role of Latino culture as a determinant of health and thus erases the adverse effects of socioeconomic status, structural racism, and its consequent embeddedness in the constraints of poverty. Compromising the ability to explain these paradoxical outcomes are methodological limitations that prevent the analyses of a wide array of unexplored yet critical factors such as demographic indicators of inequality, socioeconomic and other social determinants that can proffer deep insight and more tailored solutions to remedy health disparities in low-income communities [95–97]. We encourage researchers to critique analyses that attribute health outcomes to cultural practices and, in its stead, examine the interactions between structural and health inequities.

Research initiatives similar to Hispanic Community Health Study/Study of Latinos are integral in drawing attention to Latino heterogeneity and differential health outcomes, however the generalizability of their findings is limited as only 22.6% of the sample is U.S.-born [98]. The percent of foreign-born Latino residents has declined from 40.1% in 2000 to 32% in 2020, and current Latino population growth is driven more by new births than by new immigrants [99]. Additionally, Alzheimer’s and related dementias research has primarily been conducted with Dominican participants, despite the fact that they constitute only 3% of the U.S. population [100]. Future research should carefully consider who they recruit for their studies to ensure proportional representativeness of Latino subgroups by both intergenerational U.S. citizenship and immigration status.

Finally, studies that do examine leading causes of mortality rarely disaggregate data by key demographic SDH factors (e.g., nativity, place, sex) [19, 67, 101]. Geographic location or place reflects unique historical, socio-ethnic, and demographic contexts that may affect health across the lifespan and shape morbidity and mortality patterns [17, 102]. The Latino population in the Southwest primarily consists of Mexican-origin individuals, Puerto Ricans and Dominicans mainly reside in the Northeast, Cubans are heavily concentrated in South Florida, and large numbers of Central and South Americans live in California, Texas and Washington DC area [17]. Examining the geographic variation of Latina subgroups by country of origin will enable researchers to more accurately measure SDH and outcomes, as well as construct accurate Latina health profiles that enable the development and implementation of public health strategies, surveillance measures, and interventions that effectively address disparities.

## Conclusion

This critical analysis of the state of the science on Latina mortality for the five leading chronic diseases is a unique contribution to both the literature on Hispanic/Latino health and health disparities in general. These findings highlight the need to disentangle the subgroups of Latinas most at risk, identify conditions under which these risks increase, and provide a broader and deeper lens for epidemiologists to study mortality among differing groups of Latinas. The pan-ethnic terms Hispanic and Latina/o are insufficient for describing how multiple intersecting social determinants shape health in Latino population subgroups.

We identified critical data gaps that hinder progress in understanding sex differences within and across Latino subgroups and the examination of differences by critical demographic identifiers of inequality (e.g., place, race, and SES). Limitations included the scant number of articles that examined the structural and institutional contexts that are strongly associated with Latina mortality and an inability to identify any systematic empirical model that guided studies on Latina mortality indicators. To the extent that current epidemiologists, public health, and social scientists are calling for more robust and comprehensive models to study complex social factors that play a role in disease patterns, epidemiology needs to take a more proactive role in the study of population health. Over the past 20 years, there has been an evolution of new paradigms that include important SDH, such as structural racism, environmental context, and the intersections of poverty, race, and educational level.

A paradigmatic shift allows for more comprehensive and accurate assessments of Latina health that take into consideration the intersectionality of the SDH and provides new and diverse blueprints for innovative strategies and interventions. Interventions among Latinas have traditionally focused on individual behaviors (e.g., exercise, diet) and failed to assess the role of structural determinants in community and socio-political institutional contexts. Consequently, interventions are often designed without an environmental and socioeconomic context. As a result, many interventions have failed to demonstrate long-term effectiveness and sustainability or to provide new knowledge or strategies for addressing disparities. The application of knowledge from epidemiologic research to produce, fund, and implement initiatives to address social determinants is well documented, yet there is insufficient evidence of its widespread impact. This is most evident in the results of the nationwide Healthy People Initiative in 2020, which indicate slow or undetectable improvement in the 36 leading objectives since the initiation of Healthy People in 2000. New knowledge and interventions need to reflect the paradigmatic shift toward SDH created over the past two decades. Without new theorizing to drive research designs, interventions will continue to produce ineffectual and inconclusive results and innovative approaches to salubrious communities will remain elusive.

An equally important and quite troubling implication of this analysis is the utility of research-generated data to drive policy-making decisions. Flawed data may lead to resulting policies that also are flawed, and as a result, impede rather than contribute to the improvement of the health of communities [103–105]. The triadic associations between public health data, practice, and policy are dependent on a national shift in the scientific paradigms that underlie who creates the problem, how and where problems are viewed, and who is responsible for implementing solutions. Epidemiologists and other public health researchers are positioned at the helm of translating good public health science into good public policy. NIH and other funding agencies cannot continue to fund projects that study the same problems under the same conditions and expect a different set of results. This review confirms the importance of systematically unraveling and addressing the health disparities, SDH and resultant inequities that plague the nation’s diverse communities who are in immediate need of effective preventive and primary care interventions aimed at enhancing their health and quality of life.

### Electronic supplementary material

Below is the link to the electronic supplementary material.

Supplementary Material 1

## Data Availability

The datasets used and/or analyzed during the current study available from the corresponding author on reasonable request.

## Abbreviations

CDC: Centers for Disease Control and Prevention
NIH: National Institutes of Health
NLW: Non-Latino White(s)
SDH: Social Determinants of Health
SES: Socioeconomic Status
US/U.S.: United States
