# Supplementary material for: Critical analyses of Latina mortality: disentangling the heterogeneity of ethnic origin, place, nativity, race, and socioeconomic status
Source: BMC Public Health. 2024 Jan 16;24:190. doi: 10.1186/s12889-024-17721-9 (PMC10790397; doi:10.1186/s12889-024-17721-9)
Supplement: Supplementary file 1 — Supplementary Material 1 [file 12889_2024_17721_MOESM1_ESM.docx]

Supplemental Table 1. List of included articles (n = 52)

|  | **Data Disaggregated Further By:** | | | | |
| --- | --- | --- | --- | --- | --- |
| **Study** | **Ethnic Origin** | **Place** | **Nativity** | **Race** | **SES** |
| Altekruse SF, Henley JS, Cucinelli JE, McGlynn KA. Changing hepatocellular carcinoma incidence and liver cancer mortality rates in the United States. Official journal of the American College of Gastroenterology\| ACG. 2014 Apr 1;109(4):542-53. | - | - | - | - | - |
| Ayala C, Greenlund KJ, Croft JB, Keenan NL, Donehoo RS, Giles WH, Kittner SJ, Marks JS. Racial/ethnic disparities in mortality by stroke subtype in the United States, 1995–1998. American journal of epidemiology. 2001 Dec 1;154(11):1057-63. | - | - | - | - | - |
| Ayala C, Croft JB, Greenlund KJ, Keenan NL, Donehoo RS, Malarcher AM, Mensah GA. Sex differences in US mortality rates for stroke and stroke subtypes by race/ethnicity and age, 1995–1998. Stroke. 2002 May 1;33(5):1197-201. | - | - | - | - | - |
| Borrell LN, Rodríguez-Álvarez E, Dallo FJ. Racial/ethnic inequities in the associations of allostatic load with all-cause and cardiovascular-specific mortality risk in US adults. PLoS One. 2020 Feb 13;15(2):e0228336. | - | - | - | - | - |
| Castañeda‐Avila MA, Ortiz‐Ortiz KJ, Torres‐Cintrón CR, Birmann BM, Epstein MM. Trends in cause of death among patients with multiple myeloma in Puerto Rico and the United States SEER population, 1987–2013. International journal of cancer. 2020 Jan 1;146(1):35-43. | 🗸 | - | - | - | - |
| Chang MH, Moonesinghe R, Athar HM, Truman BI. Trends in disparity by sex and race/ethnicity for the leading causes of death in the United States—1999-2010. Journal of Public Health Management and Practice. 2016 Jan 1;22:S13-24. | - | - | - | - | - |
| Chen Y, Freedman ND, Rodriquez EJ, Shiels MS, Napoles AM, Withrow DR, Spillane S, Sigel B, Perez-Stable EJ, de González AB. Trends in premature deaths among adults in the United States and Latin America. JAMA network open. 2020 Feb 5;3(2):e1921085-. | 🗸 | - | - | - | - |
| Chen C, Markossian TW, Silva A, Tarasenko YN. Epithelial ovarian cancer mortality among Hispanic women: sub-ethnic disparities and survival trend across time: an analysis of SEER 1992–2013. Cancer epidemiology. 2018 Feb 1;52:134-41. | 🗸 | - | - | - | - |
|  | **Data Disaggregated Further By:** | | | | |
| **Study** | **Ethnic Origin** | **Place** | **Nativity** | **Race** | **SES** |
| Chu KC, Miller BA, Springfield SA. Measures of racial/ethnic health disparities in cancer mortality rates and the influence of socioeconomic status. Journal of the National Medical Association. 2007 Oct;99(10):1092. | 🗸 | - | 🗸 | - | - |
| ^a^ Coker AL, DeSimone CP, Eggleston KS, White AL, Williams M. Ethnic disparities in cervical cancer survival among Texas women. Journal of Women's Health. 2009 Oct 1;18(10):1577-83. | 🗸 | - | - | - | - |
| Corona E, Yang L, Esrailian E, Ghassemi KA, Conklin JL, May FP. Trends in esophageal cancer mortality and stage at diagnosis by race and ethnicity in the United States. Cancer Causes & Control. 2021 Aug;32(8):883-94. | - | - | - | - | - |
| Curtin SC. Trends in cancer and heart disease death rates among adults aged 45–64: United States, 1999–2017. | - | - | - | - | - |
| ^a^ de Grubb MC, Kilbourne B, Kihlberg C, Levine RS, Hood DB. Demographic and geographic variations in breast cancer mortality among US Hispanics. Journal of health care for the poor and underserved. 2013;24(1):140-52. | - | 🗸 | - | 🗸 | 🗸 |
| ^a^ Eggleston KS, Coker AL, Williams M, Tortolero-Luna G, Martin JB, Tortolero SR. Cervical cancer survival by socioeconomic status, race/ethnicity, and place of residence in Texas, 1995–2001. Journal of Women's Health. 2006 Oct 1;15(8):941-51. | - | - | - | - | 🗸 |
| ^a^ Gomez, S. L., O'Malley, C. D., Stroup, A., Shema, S. J., & Satariano, W. A. (2007). Longitudinal, population-based study of racial/ethnic differences in colorectal cancer survival: impact of neighborhood socioeconomic status, treatment and comorbidity. BMC Cancer, 7, 193. | - | - | - | - | - |
| Harper S, Lynch J, Meersman SC, Breen N, Davis WW, Reichman MC. Trends in area-socioeconomic and race-ethnic disparities in breast cancer incidence, stage at diagnosis, screening, mortality, and survival among women ages 50 years and over (1987-2005). Cancer Epidemiology Biomarkers & Prevention. 2009 Jan 1;18(1):121-31. | - | 🗸 | - | - | - |
| Hendrick RE, Monticciolo DL, Biggs KW, Malak SF. Age distributions of breast cancer diagnosis and mortality by race and ethnicity in US women. Cancer. 2021 Dec 1;127(23):4384-92. | - | - | - | - | - |
|  | **Data Disaggregated Further By:** | | | | |
| **Study** | **Ethnic Origin** | **Place** | **Nativity** | **Race** | **SES** |
| Howrey B, Goodwin JS, Eschbach K, Freeman J. Lower stroke mortality among Hispanics: an exploration of potential methodological confounders. Medical care. 2010 Jun;48(6):534. | - | - | 🗸 | - | - |
| Ingram DD, Montresor-Lopez JA. Differences in stroke mortality among adults aged 45 and over: United States, 2010-2013. US Department of Health and Human Services, Centers for Disease Control and Prevention, National Center for Health Statistics; 2015 Jul 1. | - | - | - | - | - |
| Islami F, Ward EM, Jacobs EJ, Ma J, Goding Sauer A, Lortet-Tieulent J, Jemal A. Potentially preventable premature lung cancer deaths in the USA if overall population rates were reduced to those of educated whites in lower-risk states. Cancer Causes & Control. 2015 Mar;26:409-18. | - | 🗸 | - | - | 🗸 |
| ^a^ Keegan TH, Quach T, Shema S, Glaser SL, Gomez SL. The influence of nativity and neighborhoods on breast cancer stage at diagnosis and survival among California Hispanic women. BMC cancer. 2010 Dec;10(1):1-1. | - | 🗸 | 🗸 | - | - |
| Loomis D, Schulz M. Mortality from six work‐related cancers among African Americans and Latinos. American journal of industrial medicine. 2000 Nov;38(5):565-75. | - | - | - | - | - |
| ^a^ McCarthy AM, Dumanovsky T, Visvanathan K, Kahn AR, Schymura MJ. Racial/ethnic and socioeconomic disparities in mortality among women diagnosed with cervical cancer in New York City, 1995–2006. Cancer causes & control. 2010 Oct;21:1645-55. | 🗸 | - | - | - | 🗸 |
| Mercado CI, Yang Q, Ford ES, Gregg E, Valderrama AL. Gender‐and race‐specific metabolic score and cardiovascular disease mortality in adults: A structural equation modeling approach—United States, 1988‐2006. Obesity. 2015 Sep;23(9):1911-9. | - | - | - | - | - |
| Micha R, Peñalvo JL, Cudhea F, Imamura F, Rehm CD, Mozaffarian D. Association between dietary factors and mortality from heart disease, stroke, and type 2 diabetes in the United States. Jama. 2017 Mar 7;317(9):912-24. | - | - | - | - | - |
| Narod SA, Iqbal J, Giannakeas V, Sopik V, Sun P. Breast cancer mortality after a diagnosis of ductal carcinoma in situ. JAMA oncology. 2015 Oct 1;1(7):888-96. | - | - | - | - | - |
| ^a^ Parise, C. A., & Caggiano, V. (2013). Disparities in race/ethnicity and socioeconomic status: risk of mortality of breast cancer patients in the California Cancer Registry, 2000-2010. BMC Cancer, 13, 449. doi:10.1186/1471-2407-13-449 | - | - | - | - | 🗸 |
|  | **Data Disaggregated Further By:** | | | | |
| **Study** | **Ethnic Origin** | **Place** | **Nativity** | **Race** | **SES** |
| ^a^ Patel MI, Schupp CW, Gomez SL, Chang ET, Wakelee HA. How do social factors explain outcomes in non–small-cell lung cancer among Hispanics in California? Explaining the Hispanic paradox. Journal of Clinical Oncology. 2013 Oct 10;31(28):3572. | - | - | 🗸 | - | 🗸 |
| Patel MI, Wang A, Kapphahn K, Desai M, Chlebowski RT, Simon MS, Bird CE, Corbie-Smith G, Gomez SL, Adams-Campbell LL, Cote ML. Racial and ethnic variations in lung cancer incidence and mortality: results from the Women’s Health Initiative. Journal of Clinical Oncology. 2016 Feb 2;34(4):360. | - | - | - | - | - |
| Pathak EB. Is heart disease or cancer the leading cause of death in United States women?. Women's Health Issues. 2016 Nov 1;26(6):589-94. | - | - | - | - | - |
| Philips Jr BU, Belasco E, Markides KS, Gong G. Socioeconomic deprivation as a determinant of cancer mortality and the Hispanic paradox in Texas, USA. International Journal for Equity in Health. 2013 Dec;12:1-9. | - | - | - | - | 🗸 |
| Pinheiro PS, Medina HN, Callahan KE, Koru-Sengul T, Sharma J, Kobetz EN, Penedo FJ. Kidney cancer mortality disparities among Hispanics in the US. Cancer epidemiology. 2021 Jun 1;72:101938. | 🗸 | - | - | - | - |
| Rauh-Hain JA, Melamed A, Schaps D, Bregar AJ, Spencer R, Schorge JO, Rice LW, Del Carmen MG. Racial and ethnic disparities over time in the treatment and mortality of women with gynecological malignancies. Gynecologic oncology. 2018 Apr 1;149(1):4-11. | - | - | - | - | - |
| Rodriguez F, Hastings KG, Boothroyd DB, Echeverria S, Lopez L, Cullen M, Harrington RA, Palaniappan LP. Disaggregation of cause-specific cardiovascular disease mortality among Hispanic subgroups. JAMA cardiology. 2017 Mar 1;2(3):240-7. | 🗸 | - | - | - | - |
| San Miguel Y, Gomez SL, Murphy JD, Schwab RB, McDaniels-Davidson C, Canchola AJ, Molinolo AA, Nodora JN, Martinez ME. Age-related differences in breast cancer mortality according to race/ethnicity, insurance, and socioeconomic status. BMC cancer. 2020 Dec;20:1-9. | - | - | - | - | - |
| Savage K, Williams JS, Garacci E, Egede LE. Association between cardiovascular disease risk factors and mortality in adults with diabetes: a stratified analysis by sex, race, and ethnicity. International Journal of Public Health. 2022 Apr 6;67:1604472. | - | - | - | - |  |
|  | **Data Disaggregated Further By:** | | | | |
| **Study** | **Ethnic Origin** | **Place** | **Nativity** | **Race** | **SES** |
| Sharma S, Vik S, Pakseresht M, Shen L, Kolonel LN. Diet impacts mortality from cancer: results from the multiethnic cohort study. Cancer Causes & Control. 2013 Apr;24:685-93. | - | - | - | - | - |
| ^a^ Shariff-Marco S, Yang J, John EM, Sangaramoorthy M, Hertz A, Koo J, Nelson DO, Schupp CW, Shema SJ, Cockburn M, Satariano WA. Impact of neighborhood and individual socioeconomic status on survival after breast cancer varies by race/ethnicity: the Neighborhood and Breast Cancer Study. Cancer epidemiology, biomarkers & prevention. 2014 May 1;23(5):793-811. | - | - | - | - | 🗸 |
| ^a^ Sheppard CS, El‐Zein M, Ramanakumar AV, Ferenczy A, Franco EL. Assessment of mediators of racial disparities in cervical cancer survival in the United States. International journal of cancer. 2016 Jun 1;138(11):2622-30. | - | 🗸 | - | - | 🗸 |
| Shiels MS, Haque AT, Haozous EA, Albert PS, Almeida JS, García-Closas M, Nápoles AM, Pérez-Stable EJ, Freedman ND, Berrington de González A. Racial and ethnic disparities in excess deaths during the COVID-19 pandemic, March to December 2020. Annals of internal medicine. 2021 Dec;174(12):1693-9. | - | - | - | - | - |
| Siegel RL, Sahar L, Portier KM, Ward EM, Jemal A. Cancer death rates in US congressional districts. CA: a cancer journal for clinicians. 2015 Sep;65(5):339-44. | - | - | - | - | - |
| Simard EP, Fedewa S, Ma J, Siegel R, Jemal A. Widening socioeconomic disparities in cervical cancer mortality among women in 26 states, 1993‐2007. Cancer. 2012 Oct 15;118(20):5110-6. | - | - | - | - | 🗸 |
| Singh GK, Jemal A. Socioeconomic and racial/ethnic disparities in cancer mortality, incidence, and survival in the United States, 1950–2014: over six decades of changing patterns and widening inequalities. Journal of environmental and public health. 2017 Oct;2017. | - | - | - | - | - |
| Singh GK, Siahpush M, Altekruse SF. Time trends in liver cancer mortality, incidence, and risk factors by unemployment level and race/ethnicity, United States, 1969–2011. Journal of community health. 2013 Oct;38:926-40. | - | - | - | - | 🗸 |
| ^a^ Singh GK, Hiatt RA. Trends and disparities in socioeconomic and behavioural characteristics, life expectancy, and cause-specific mortality of native-born and foreign-born populations in the United States, 1979–2003. International journal of epidemiology. 2006 Aug 1;35(4):903-19. | - | - | 🗸 | - | - |
|  | **Data Disaggregated Further By:** | | | | |
| **Study** | **Ethnic Origin** | **Place** | **Nativity** | **Race** | **SES** |
| Singh GK, Siahpush M. All-cause and cause-specific mortality of immigrants and native born in the United States. American journal of public health. 2001 Mar;91(3):392. | - | - | - | - | - |
| Smith CA, Barnett E. Diabetes-related mortality among Mexican Americans, Puerto Ricans, and Cuban Americans in the United States. Revista panamericana de salud pública. 2005 Dec;18(6):381-7. | 🗸 | - | - | - | - |
| Soto‐Salgado M, Suárez E, Calo W, Cruz‐Correa M, Figueroa‐Vallés NR, Ortiz AP. Incidence and mortality rates for colorectal cancer in Puerto Rico and among Hispanics, non‐Hispanic whites, and non‐Hispanic blacks in the United States, 1998‐2002. Cancer. 2009 Jul 1;115(13):3016-23. | 🗸 | - | - | - | - |
| Tejada-Vera B. Mortality from Alzheimer's disease in the United States: data for 2000 and 2010. US Department of Health and Human Services, Centers for Disease Control and Prevention, National Center for Health Statistics; 2013. | - | - | - | - | - |
| ^a^ Tian N, Goovaerts P, Zhan FB, Chow TE, Wilson JG. Identifying risk factors for disparities in breast cancer mortality among African-American and Hispanic women. Women's Health Issues. 2012 May 1;22(3):e267-76. | - | 🗸 | - | - | - |
| Zambrana RE, Ayala C, Pokras OC, Minaya J, Mensah GA. Disparities in hypertension-related mortality among selected Hispanic subgroups and non-Hispanic white women ages 45 years and older–united states, 1995–1996 and 2001–2002. Ethnicity & Disease. 2007 Jul 1;17(3):434-40. | 🗸 | - | - | - | - |
| Zheng ZJ, Croft JB, Giles WH, Mensah GA. Sudden cardiac death in the United States, 1989 to 1998. Circulation. 2001 Oct 30;104(18):2158-63. | - | - | - | - | - |

^a^ indicates that article was found via lateral search strategy.
